# Supplementary material for: Determinants of transcription factor regulatory range
Source: Nat Commun. 2020 May 18;11:2472. doi: 10.1038/s41467-020-16106-x (PMC7235260; doi:10.1038/s41467-020-16106-x)
Supplement: Supplementary file 1 — Supplementary Information [file 41467_2020_16106_MOESM1_ESM.pdf]

Supplementary Information

Determinants of transcription factor regulatory range

Chen et al.

Supplementary Figures:

Supplementary Figure 1
Supplementary Figure 2
Supplementary Figure 3
Supplementary Figure 4
Supplementary Figure 5

Supplementary Figure 1

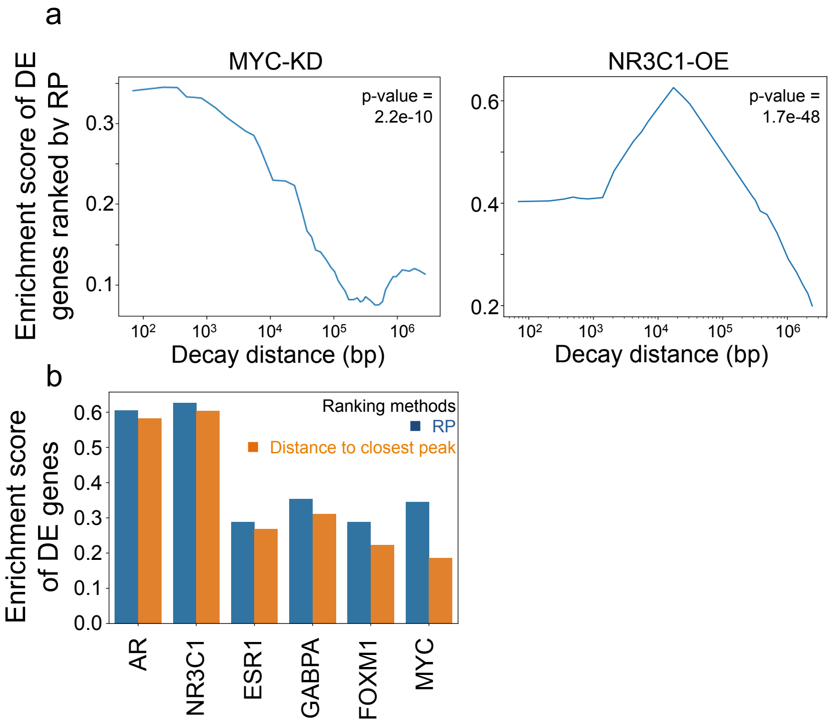

Supplementary Figure 1: Inferring TF regulatory ranges using TF perturbation data and TF-ChIP-seq reveals two distinct TF classes

(a) Inferences of TF i-specific regulatory decay distances ($\Delta_i{}^*$), defined as the decay distance (Δ) that can best separate TF i perturbation-induced differentially expressed (DE) gene sets from other genes. The regulatory potential, $R_{i,j}(\Delta)$, parametrized with a short-range Δ (<1kb) best separates the MYC-knockdown DE gene set from other genes (left). On the contrary, the NR3C1-overexpressed DE gene set is best separated from other genes by $R_{i,j}(\Delta)$ with a long-range Δ (>10kb). The two-sided Kolmogorov–Smirnov two-sample test is used to estimate the degree of separation of DE genes from other genes.

(b) Comparison of the RP model performance with the closest peak heuristic. Models were evaluated using ChIP-seq peak data combined with down-regulated DE genes upon knock-down of MYC, FOXM1, GABPA, ESR1; and up-regulated DE genes on stimulation of AR (DHT stimulation), and NR3C1 (Dexamethasone treatment). Genes are ranked based on either RP scores or TSS-to-closest-TF-peak-distances, and the Two-sided Kolmogorov–Smirnov two-sample test is used to evaluate the separation of DE genes from other genes.

Supplementary Figure 2

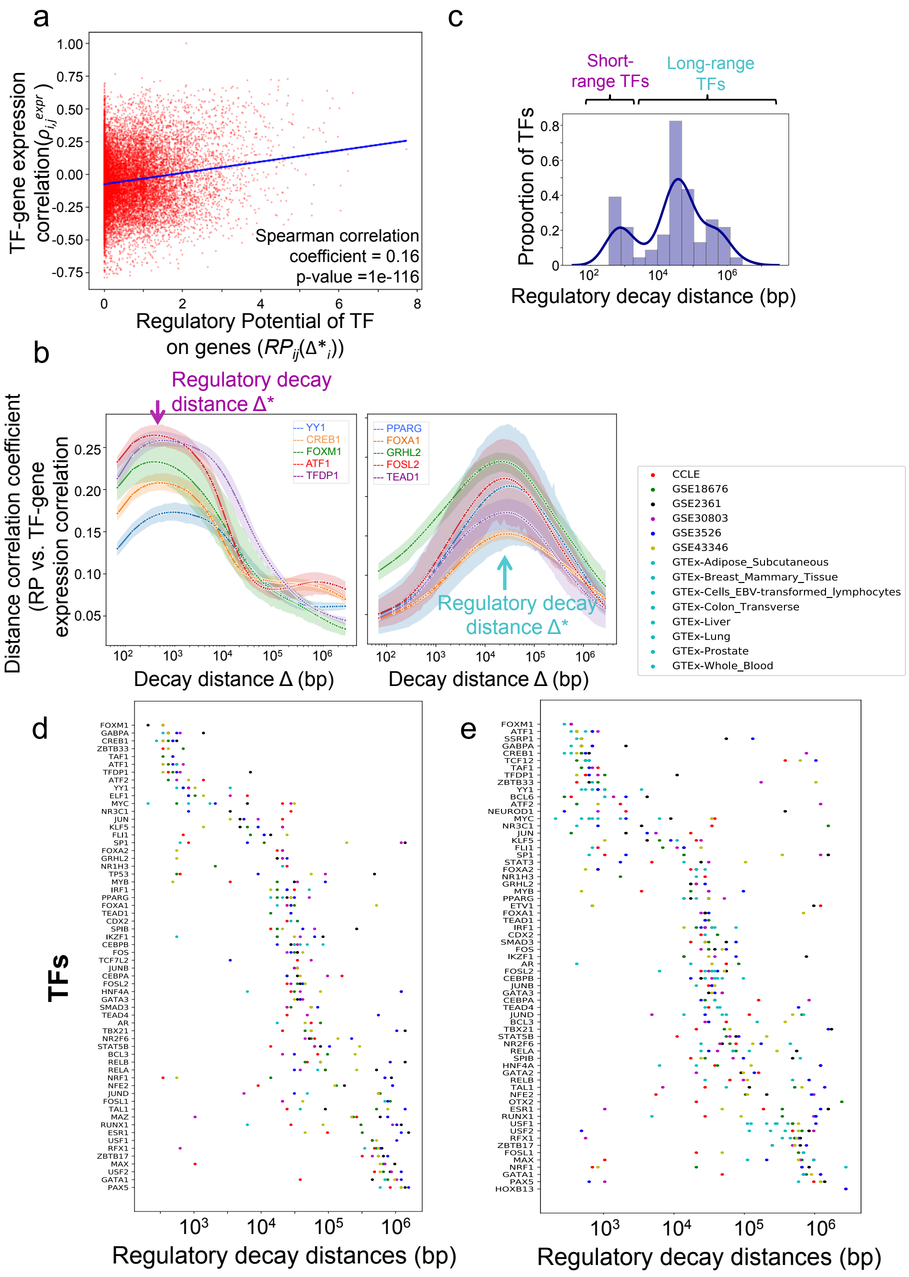

Supplementary Figure 2 (cont.)

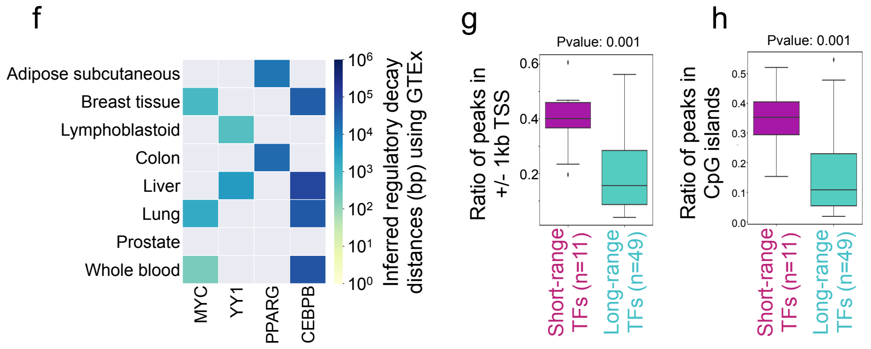

Supplementary Figure 2: Inferring TF regulatory ranges using gene expression cohorts and TF-ChIP-seq reveals two distinct TF classes

(a) Correlation of TF i – gene j expression correlations ($\rho_{i,j}^{\mathrm{expr}}$) and regulatory potential $R_{i,j}(\Delta_i{}^*)$. Each dot represents one gene, and the straight line represents a linear relationship between $\rho_{i,j}^{\mathrm{expr}}$ and $R_{i,j}(\Delta_i{}^*)$. The p-value is calculated using the Spearman correlation.

(b) Inference of regulatory decay distances ($\Delta_i{}^*$) using distance correlation as the measure of goodness of fit. Representative TFs with short-range $\Delta_i{}^*$ (100bp-3kb) include YY1, CREB1, FOXM1, ATF1, and TFDP1 (left). Representative TFs with long-range $\Delta_i{}^*$ (3 kb-100 kb) include PPARG, FOXA1, GRHL2, FOSL2, and TEAD1 (right). The colored shaded regions depict the 95% confidence intervals derived from all ChIP-seq samples that passed QC for each single TF. Dots along the line represent the evaluated Δ values.

(c) Distribution of regulatory decay distances ($\Delta_i{}^*$) of 65 TFs using distance correlation as the goodness of fit measure. 14 TFs are short-range (left), 51 TFs are long-range (right).

(d-e) Inferred TF regulatory decay distances ($\Delta_i{}^*$) are consistent across CCLE, GTEx, and other 5 gene expression cohorts using linear (d) and distance correlation measure (e).

(f) For a given TF, tissue-specific $\Delta_i{}^*$ are similar across different tissues. X-axis: TFs; Y-axis: GTEx tissues. Inferred tissue-specific $\Delta_i{}^*$ are color coded in entries.

(g-h) Short-range TFs (magenta) are more likely to be located within 1kb of TSS (g) or CpG islands (h) compared to long-range ones (green). The p-value was calculated using the two-sided Student's t-test. The box plots extend from the lower to the upper quartile values of the data, with a line at the median. The whiskers extend from the box to show the range of the data.

Supplementary Figure 3

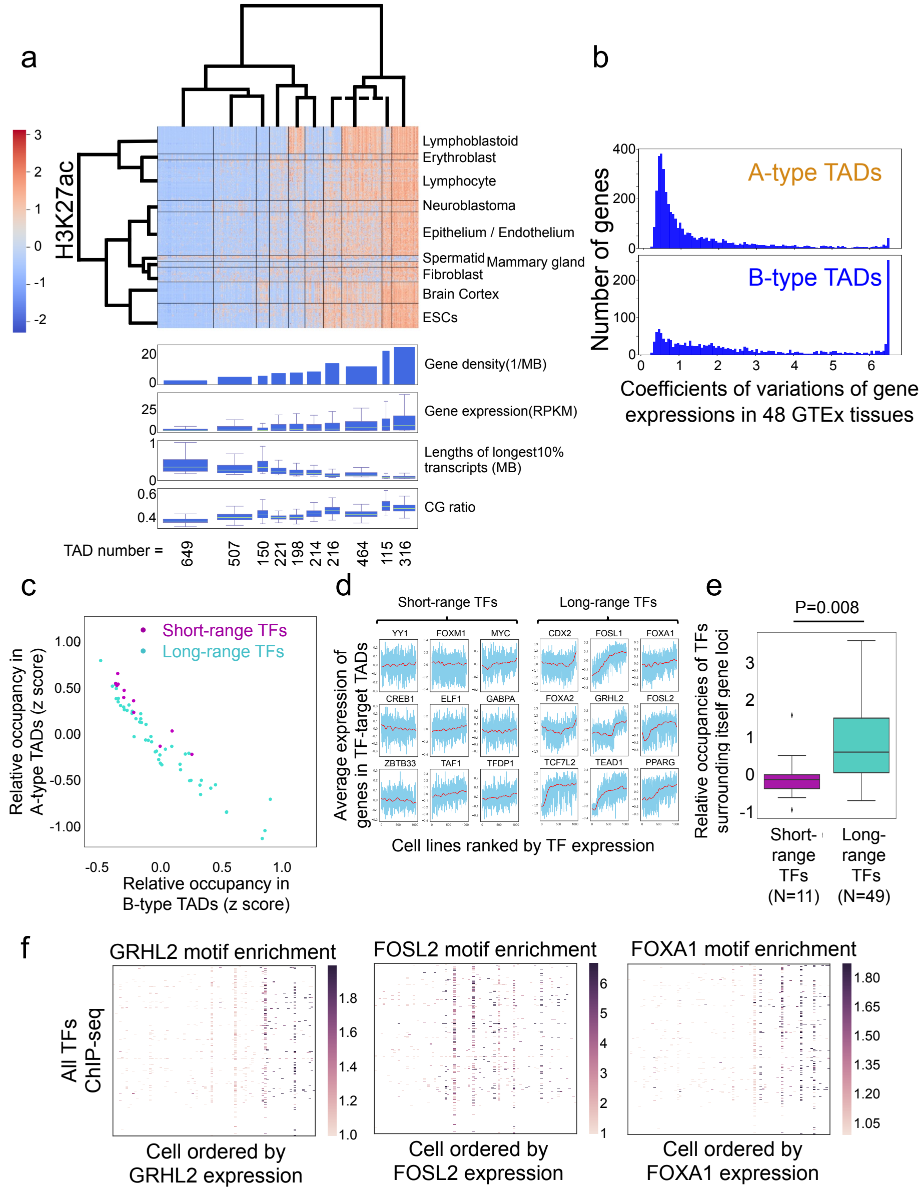

Supplementary Figure 3: Long-range and short-range TFs have distinct regulatory properties.

(a) Gene densities, average gene expression, lengths of the longest 10% transcripts, and G/C ratios correspond to the H3K27ac levels. The box plots extend from the lower to the upper quartile values of the data, with a line at the median. The whiskers extend from the box to show the range of the data.

(b) Genes in B-type TADs have more tissue-restricted gene expression levels, indicated by higher expression coefficients of variation (i.e., ratio of the standard deviation to the mean) across 48 GTEx tissues. x-axis: gene expression coefficients of variation. y-axis: gene numbers.

(c) Relative TF occupancies in A-type and B-type TADs. As a trend, the short-range TFs have higher relative occupancies than long-range TFs in A-type TADs, and a large proportion of long-range TFs are enriched in B-type TADs.
(d) Right: In cell lines, the average expression of genes located within long-range TF target TADs increases with the level of expression of the long-range TFs themselves. Left: The same trend does not appear for short-range TFs.
(e) Relative occupancies (z-scores) of TF ChIP-seq peaks within the TAD harboring the TF gene itself. The p-value was calculated using the two-sided Student t-test. The box plots extend from the lower to the upper quartile values of the data, with a line at the median. The whiskers extend from the box to show the range of the data.
(f) Increasing enrichment of long-range TF *i* (Left to right: GRHL2, FOSL2, FOXA1) motif in the ChIP-seq binding sites of TFs besides TF *i* in cells with high expression of TF *i*. x-axis: ChIP-seq cell lines ranked by TF *i* expression using CCLE expression data. y-axis: ChIP-seq of other TFs. Each entry represents the TF *i* motif enrichment in ChIP-seq peaks of the corresponding TF-cell sample. Gray cells in the heatmap indicate the corresponding TF-cell ChIP-seq samples that are not available.

Supplementary Figure 4

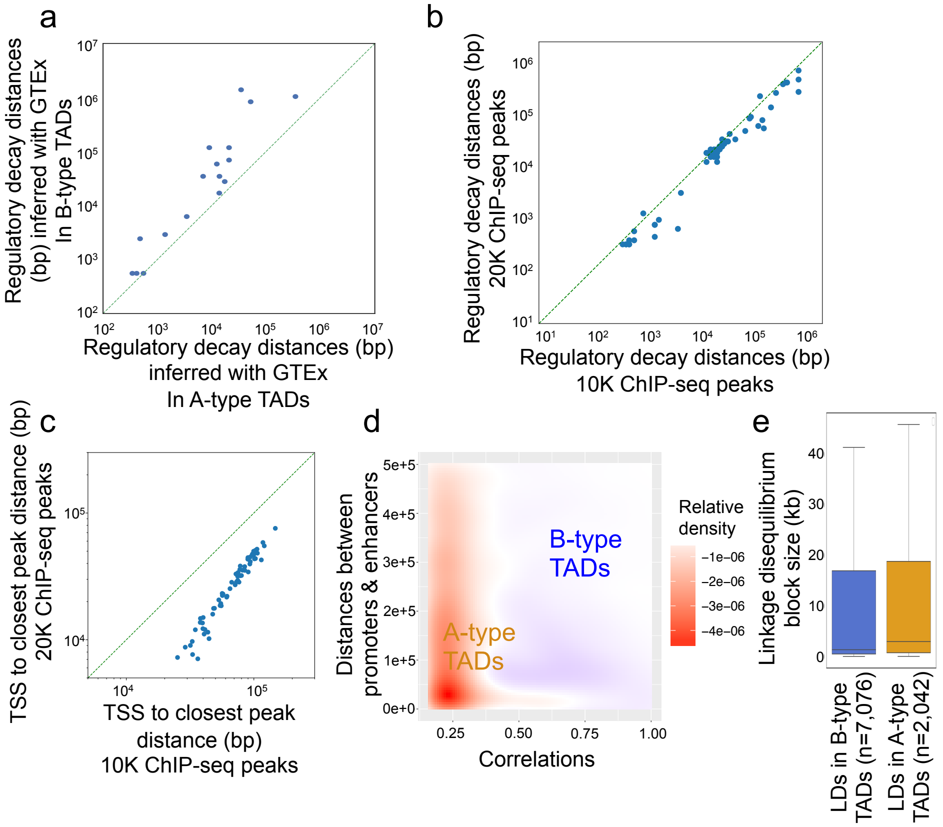

Supplementary Figure 4: Long-range TFs have longer regulatory decay distances in B-type TADs

(a) Longer TF regulatory decay distances in B-type TADs using GTEx gene expression data. x-axis: inferred regulatory decay distances in A-type TADs. y-axis: inferred regulatory decay distances in B-type TADs.

(b) Regulatory decay distances inferred using the most significant 10,000 and 20,000 peaks are highly consistent (Two-sided Pearson correlation p-value $10e^{-38}$).

(c) The average distances of closest TF-peak to TSS halve if increasing the ChIP-seq peak number from 10,000 to 20,000.

(d) Relative density map of distances and expression correlations between mRNAs and eRNAs measured in A-type and B-type TADs using cap analysis gene expression (CAGE-seq). Red: higher density of mRNAs - eRNAs pairs in A-type TADs compared with B-type TADs. Purple: higher density of mRNAs - eRNAs pairs in B-type TADs compared with A-type TADs.

(e) Linkage disequilibrium block sizes in A-type TADs (Red) and B-type TADs (Blue) do not explain the eQTL-to-TSS distance differences between A-type and B-type TADs. The box plots extend from the lower to the upper quartile values of the data, with a line at the median. The whiskers extend from the box to show the range of the data.

Supplementary Figure 5

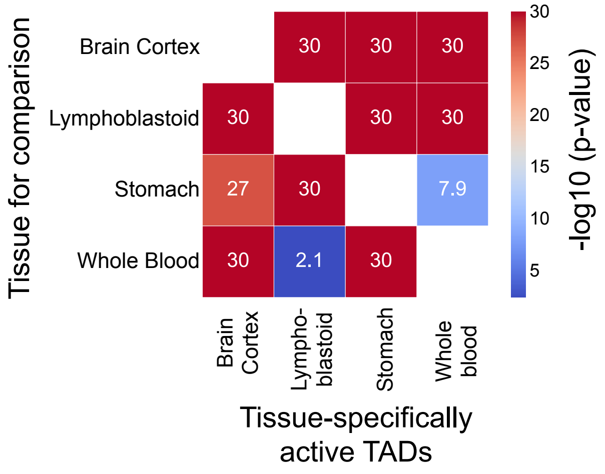

Supplementary Figure 5: Regulatory decay distance changes with TAD activity and chromatin state

A generalization of Fig. 5c: GTEx eQTL-TSS distances where compared in TADs that showed tissue-restricted activities. 12 comparisons were made between four tissues: brain cortex, lymphoblastoid, stomach, and whole blood. For each tissue pair defined by row *i* and column *j*, TADs with higher H3K27ac levels in tissue *j* than tissue *i* were identified. In these differentially active TADs the eQTL-TSS distances of eQTLs identified in tissue *i* were compared with the eQTL-TSS distances of eQTLs identified in tissue *j*. The color of the cell *i,j* represents the $\log_{10}$ one-sided Student's t-test p-value measure of significance. In 10/12 comparisons the GTEx eQTL-TSS distances change as expected: they are significantly shorter in the TADs that are active in the tissues in which the eQTL is measured.
