# Supplementary material for: Determinants of transcription factor regulatory range
Source: Nat Commun. 2020 May 18;11:2472. doi: 10.1038/s41467-020-16106-x (PMC7235260; doi:10.1038/s41467-020-16106-x)
Supplement: Supplementary file 3 — Description of Additional Supplementary Information [file 41467_2020_16106_MOESM3_ESM.pdf]

Description of Additional Supplementary Files

File Name: Supplementary Data 1
Description: TF_regulatory_distance: Inferred TF regulatory distances using multiple expression cohorts.

File Name: Supplementary Data 2
Description: hg38_H1_TAD_mean_H3K27ac.txt_normalized: Mean H3K27ac level in TADs across multiple cell types and tissues.

File Name: Supplementary Data 3
Description: TAD_cluster_annotation: Coordinates and cluster assignment of TADs.

File Name: Supplementary Data 4
Description: TAD_cluster_GO: Gene ontologies of genes in TAD clusters.

File Name: Supplementary Data 5
Description: Unnormalized_TF_occupancy_in_TADs: TF ChIP-seq peak densities in TADs.

File Name: Supplementary Data 6
Description: TF_target_TAD_cluster_GO: Gene ontologies of genes in TF-target TADs.

File Name: Supplementary Data 7
Description: Pioneer_like_factors: Inferred TFs with or without pioneer factor-like properties.
