# Supplementary material for: Determinants of transcription factor regulatory range
Source: Nat Commun. 2020 May 18;11:2472. doi: 10.1038/s41467-020-16106-x (PMC7235260; doi:10.1038/s41467-020-16106-x)
Supplement: Supplementary file 11 — Reporting Summary [file 41467_2020_16106_MOESM11_ESM.pdf]

nature research

Corresponding author(s): X. Shirley Liu
Last updated by author(s): 2020/3/2

Reporting Summary

Nature Research wishes to improve the reproducibility of the work that we publish. This form provides structure for consistency and transparency in reporting. For further information on Nature Research policies, see Authors & Referees and the Editorial Policy Checklist .

Statistics

For all statistical analyses, confirm that the following items are present in the figure legend, table legend, main text, or Methods section.

n/a	Confirmed	
☐	☒	The exact sample size (*n*) for each experimental group/condition, given as a discrete number and unit of measurement
☐	☒	A statement on whether measurements were taken from distinct samples or whether the same sample was measured repeatedly
☐	☒	The statistical test(s) used AND whether they are one- or two-sided<br>*Only common tests should be described solely by name; describe more complex techniques in the Methods section.*
☐	☒	A description of all covariates tested
☐	☒	A description of any assumptions or corrections, such as tests of normality and adjustment for multiple comparisons
☐	☒	A full description of the statistical parameters including central tendency (e.g. means) or other basic estimates (e.g. regression coefficient) AND variation (e.g. standard deviation) or associated estimates of uncertainty (e.g. confidence intervals)
☐	☒	For null hypothesis testing, the test statistic (e.g. *F*, *t*, *r*) with confidence intervals, effect sizes, degrees of freedom and *P* value noted<br>*Give P values as exact values whenever suitable.*
☒	☐	For Bayesian analysis, information on the choice of priors and Markov chain Monte Carlo settings
☐	☒	For hierarchical and complex designs, identification of the appropriate level for tests and full reporting of outcomes
☐	☒	Estimates of effect sizes (e.g. Cohen's *d*, Pearson's *r*), indicating how they were calculated

Our web collection on statistics for biologists contains articles on many of the points above.

Software and code

Policy information about availability of computer code

Data collection	All data are from public domain. No special software was used for data collection.
Data analysis	Open source software are listed below:<br>MACS2 2.1.2: https://github.com/taoliu/MACS<br>LiftOver: http://hgdownload.soe.ucsc.edu/admin/exe/macOSX.x86_64/liftOver<br>Homer v4.11: http://homer.ucsd.edu/homer/index.html<br><br>For inference of TF regulatory decay distance: https://bitbucket.org/liulab/tf_regulatory_distance/src/master/<br>Scripts specific for each figure are available with request to corresponding authors.

For manuscripts utilizing custom algorithms or software that are central to the research but not yet described in published literature, software must be made available to editors/reviewers. We strongly encourage code deposition in a community repository (e.g. GitHub). See the Nature Research guidelines for submitting code & software for further information.

Data

Policy information about availability of data

All manuscripts must include a data availability statement. This statement should provide the following information, where applicable:

- Accession codes, unique identifiers, or web links for publicly available datasets
- A list of figures that have associated raw data
- A description of any restrictions on data availability

Public data:
Cistrome: http://cistrome.org/db/#/

CCLE: https://portals.broadinstitute.org/ccle
GTEx: https://www.gtexportal.org/home/
Hi-C: ftp://cooler.csail.mit.edu/coolers/hg19/
GWAS: https://www.ebi.ac.uk/gwas/home
Linkage disequilibrium block: http://distild.jensenlab.org/download.html
TAD annotation: http://chromosome.sdsc.edu/mouse/hi-c/hESC.domain.tar.gz
CAGE: http://enhancer.binf.ku.dk/presets/

The processed data underling Figures 1e, 2a, 2b, 2c, 3h, and Supplementary Figure 2d are provided as Supplementary Data. Table for all figures are provided as Source Data.

Field-specific reporting

Please select the one below that is the best fit for your research. If you are not sure, read the appropriate sections before making your selection.

- [x] Life sciences
- [ ] Behavioural & social sciences
- [ ] Ecological, evolutionary & environmental sciences

For a reference copy of the document with all sections, see nature.com/documents/nr-reporting-summary-flat.pdf

Life sciences study design

All studies must disclose on these points even when the disclosure is negative.

Sample size	We included all the data without predefining sample size.
Data exclusions	For each TF ChIPseq, we included the top 20,000 peaks based on peak intensity for fair peak density comparison. Such choice did not change the result, since using top 10,000 peaks give rise to the same inference of TF regulatory ranges.
Replication	We used totally 7 gene expression cohort and inferred the TF regulatory ranges independently.
Randomization	Randomization is not applicable to this study since it did not separate the sample into different groups.
Blinding	Randomization is not applicable to this study since it did not separate the sample into different groups.

Reporting for specific materials, systems and methods

We require information from authors about some types of materials, experimental systems and methods used in many studies. Here, indicate whether each material, system or method listed is relevant to your study. If you are not sure if a list item applies to your research, read the appropriate section before selecting a response.

Materials & experimental systems

n/a	Involved in the study	
x		Antibodies
x		Eukaryotic cell lines
x		Palaeontology
x		Animals and other organisms
x		Human research participants
x		Clinical data

Methods

n/a	Involved in the study	
x		ChIP-seq
x		Flow cytometry
x		MRI-based neuroimaging
